# Effect of specimen processing, growth supplement, and different metabolic population on *Mycobacterium tuberculosis* laboratory diagnosis

**DOI:** 10.1371/journal.pone.0230927

**Published:** 2020-04-03

**Authors:** Shashikant Srivastava, Moti Chapagain, Tawanda Gumbo

**Affiliations:** Center for Infectious Diseases Research and Experimental Therapeutics, Baylor Institute for Immunology Research, Dallas, Texas, United States of America; Rutgers Biomedical and Health Sciences, UNITED STATES

## Abstract

**Introduction:**

Sputum specimen decontamination steps are essential due to the presence of other saprophytic and infectious organisms. However, they negatively affect the mycobacterial recovery. In addition, little is known about the *Mycobacterium* tuberculosis killing efficacy of the PANTA (polymyxin-B, amphotericin-B, nalidixic acid, trimethoprim, azilocillin) antibiotics. Moreover, *M*. *tuberculosis* can be present in more than one metabolic population, but the effect of different growth characteristics on the mycobacterial growth indicator tube (MGIT) based time-to-positive (TTP) is not well studied.

**Methods:**

We performed—(1) experiments using the solid agar and MGIT method to determine the effect of the NALC-NaOH decontamination method, (2) concentration-response studies with each individual antibiotic in the PANTA, and (3) the effect of the *M*. *tuberculosis* metabolic population on the TTP. TTP was recorded using the Epicenter software and exponential growth equation was used to calculate the doubling time of the bacteria, whereas, CFU/mL was analyzed using the Inhibitory Sigmoid E_max_ model for each antibiotic.

**Results:**

Decontamination resulted in 4.36+0.13 log_10_ CFU/mL difference in cultures treated with NALC-NaOH versus no decontamination process and the limit of detection decreased from 1.47 log_10_ CFU/mL to the 0.42 log_10_ CFU/mL following NALC-NaOH treatment. PANTA at currently used antibiotic concentrations, did not had negative effect on mycobacterial recovery. Exponential growth model estimated doubling time for the log-phase growth *M*. *tuberculosis* as 2.04 days, for the semi-dormant bacilli as 2.80 days, and 6.37 days for the anaerobic cultures.

**Conclusion:**

Specimen decontamination method negatively affect the laboratory diagnosis of *M*. *tuberculosis*, polymyxin-B and nalidixic acid have anti-tuberculosis efficacy at high concentrations, and the doubling time of different metabolic population should be considered when deciding the time-in-protocol for the MGIT system.

## Introduction

Several diagnostic techniques including microscopy, culture, and molecular probes are used to evaluate the presence of mycobacteria in respiratory specimens, where each technique has its advantages and disadvantages.[1–5] Acid-fast staining is technically simple, rapid, inexpensive, and highly specific but lacks sensitivity. The culture-based methods are highly sensitive but susceptible to contamination problems and therefore, subjected to decontamination steps prior to inoculation of agar or liquid culture media that adds inaccuracy as some mycobacteria die during the specimen processing. However, the specimen decontamination steps are essential due to the presence of other saprophytic and infectious organisms in specimens. Molecular techniques are the most sensitive among the diagnostic methods as they do not detect the viable bacteria, but still are expensive and sometimes technically complex.

The NALC-NaOH based specimen decontamination method is the most commonly used.[5] However, it has been shown to negatively affect the mycobacterial recovery.[6] The loss in bacterial burden is a cause of concern, especially for the specimens collected from children where the disease is paucibacillary and other populations with low bacterial burden.[7] A cocktail of antibiotic, PANTA (polymyxin-B, amphotericin-B, nalidixic acid, trimethoprim, azilocillin), is used to kill other bacteria to promote mycobacterial growth.[5] But little is known if these antibiotics have killing efficacy against *Mycobacterium tuberculosis*. Similarly, *M*. *tuberculosis* can be present in more than one metabolic population,[8] but how the different growth characteristics can affect the mycobacterial growth indicator tube (MGIT) based time-to-positive (TTP) is not well studied. Here we performed a series of experiments using the solid agar and MGIT method to determine the effect of the NALC-NaOH decontamination method followed by concentration-response studies with each individual antibiotic in the PANTA, and finally the effect of the *M*. *tuberculosis* metabolic population on the TTP.

## Materials and methods

### Bacterial strains, growth conditions, reagents and supplies

Prior to each experiment stock *M*. *tuberculosis* (H37Ra ATCC#25177 and H37Rv ATCC#27294) culture was thawed and grown in Middlebrook 7H9 broth supplemented with 10% oleic acid, albumin, dextrose, and catalase (OADC) at 37ºC under 5% CO_2_ and shaking conditions. Semi-dormant bacilli (SDB) cultures (growing under acidic conditions) were generated by transforming four day old log phase growth using the methods described previously.[9] Anaerobic cultures (under hypoxia) were generated using the methods described elsewhere.[10, 11] Mycobacterial Growth Indicator Tube (MGIT) system and supplies were purchased from BD Sciences. All the drugs used in the study were purchased from Sigma (St. Louis, MO, USA).

### Sample decontamination, antibiotic supplement, and growth effect

We used the standard NALC-NaOH specimen decontamination protocol,[5] except instead of using the clinical specimen the studies were performed with axenic *M*. *tuberculosis* cultures. The final concentration of NALC and NaOH were 0.5% and 3%, respectively. Briefly, optical density of the log phase growth *M*. *tuberculosis* cultures was measured to set the inoculum bacterial burden to ~7 log_10_ CFU/mL. One portion of the culture was processed using the NALC-NaOH decontamination protocol, then serially 10-fold diluted and inoculated on the Middlebrook 7H10 agar supplemented with 10% OADC supplement, incubated at 37ºC under 5% CO_2_ for 21 days before the colonies were counted. The processed sample was also inoculated into the MGIT tubes with and without PANTA supplement to record the TTP, that was later used to determine the correlation between the bacterial burden (CFU/mL) and the TTP. The second portion of the axenic culture was not subjected to the decontamination protocol, but 10-fold serial dilutions were prepared and inoculated on the Middlebrook 7H10 agar and MGIT tubes with and without PANTA supplement to record the CFU/mL, and TTP, respectively. The antibiotic concentrations in the PANTA were as following–polymyxin-B 40000 Units/L, nalidixic acid 160 mg/L, and amphotericin-B, trimethoprim, and azilocillin each at 4 mg/L. Experiments were performed in triplicate.

### Concentration response studies with PANTA

There were eight different concentrations of each antibiotic used in the concentration-response studies, including the concentration used in the PANTA supplement. Turbidity of the log phase growth culture was adjusted to Optical Density (OD_A600_) 0.7, then cultures were back diluted 100-fold to get ~5 log_10_ CFU/mL starting bacterial burden (inoculum) and co-incubated with eight different concentrations including the non-treated controls. After 7 days of incubation at 37ºC under 5% CO_2_, cultures were washed twice with normal saline, 10-fold serial dilution was made to inoculate the agar and the MGIT tubes. *M*. *tuberculosis* colonies were recorded after 21 days of incubation, while the MGIT derived TTP was recorded using the Epicenter software. CFU/mL and TTP data was analyzed using the Inhibitory Sigmoid E_max_ model and exponential growth model, respectively (GraphPad Prism v7).

### Time-to-positive for the different *M*. *tuberculosis* metabolic populations

For the actively replicating bacteria, log phase growth cultures were prepared as described above. Semi-dormant bacterial population was generated by transforming the log-phase growth culture.[9] To achieve this, cultures were centrifuged at 1000 rpm for 5 min at room temperature, followed by resuspending the bacterial pellet in Middlebrook 7H9 acidified to pH 5.8 using citric acid and supplemented with 10% dextrose. The cultures were incubated at 37°C without shaking for 4 days, and then used in the experiments. We used the Wayne model [10] to generate the non-replicating persisters growing under hypoxic environment for 21 days. For each metabolic population, to estimate the bacterial burden and TTP, 10-fold serial dilutions were made and inoculated on the agar and the MGIT tubes, respectively.

## Results

**Fig 1:**
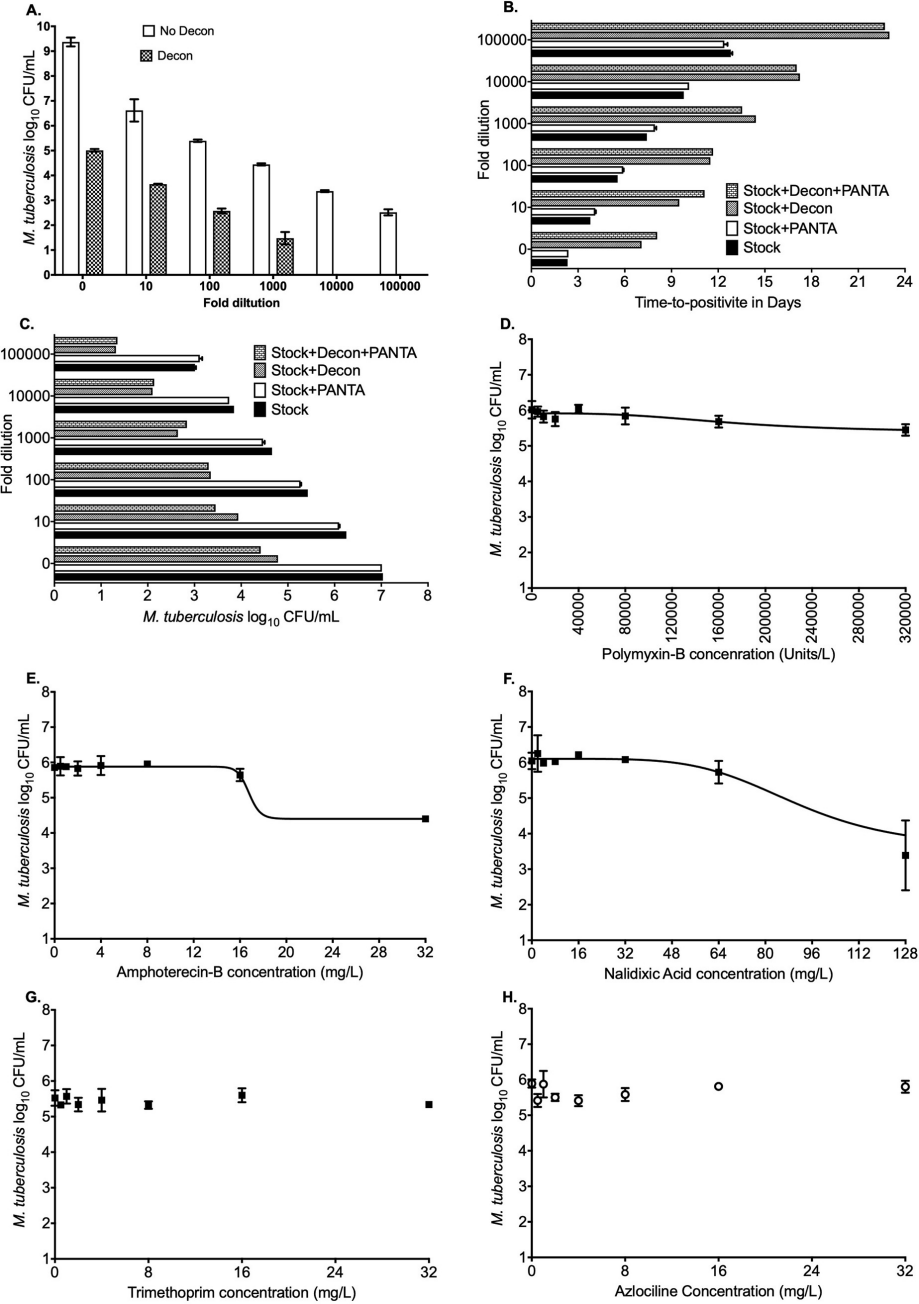
Effect of specimen processing and kill curves for the antibiotics in the PANTA supplement. **(A.)** There was 4.36+0.13 log_10_ CFU/mL difference in the bacterial burden between the cultures treated with NALC-NaOH versus no decontamination process. The limit of detection following the decontamination process was 1.47 log_10_ CFU/mL compare to the 0.42 log_10_ CFU/mL with no NALC-NaOH treatment. This loss in the bacterial burden means that specimens from children, where the disease is paucibacillary, could be erroneously flagged culture negative. Both **(B)** Time-to-positive and **(C)** solid agar methods showed that the decontamination steps significantly decrease the recovery of the *M*. *tuberculosis*. **(D-H)** Inhibitory Sigmoid E_max_ model derived kill curves with each of the five antibiotics in the PANTA supplement, where the model did not converge for trimethoprim and azilocillin. (Stock, pure culture without NALC-NaOH treatment; Decon, decontamination).

As shown in **Fig 1A**, the bacterial burden in the inoculum of the first set of experiment to determine the extent of loss of mycobacteria due to the decontamination process was 9.37+0.19 log_10_ CFU/mL. We found that the loss in the recovery of *M*. *tuberculosis* was similar across the 10-fold serially diluted samples and the limit of detection using solid agar-based method was 1.44+0.25 log_10_ CFU/mL following the NALC-NaOH treatment. **Fig 1B** and **1C** show the TTP and corresponding log_10_ CFU/mL bacteria in each of the four set of samples: no NALC-NaOH treatment (Stock), Stock plus PANTA, Stock plus NALC-NaOH treatment, and Stock plus NALC-NaOH treatment plus PANTA. These results show that while addition of antibiotics at the concentration used in the PANTA supplement do not have any effect of the bacterial growth, the decrease in the mycobacteria recovery following treatment with the NALC-NaOH was consistent with the results shown in **Fig 1A**. **Fig 1D–1H** show the results of the inhibitory Sigmoid E_max_ model for each of the five antibiotics in PANTA. None of the antibiotics killed *M*. *tuberculosis* below stasis (bacterial burden in the inoculum on day 0). Polymyxin-B showed a kill of 0.53 log_10_ CFU/mL *M*. *tuberculosis* with the highest concentration of 320000 Units/L and effective concentration (EC) mediating 50% of the maximal kill (E_max_) was calculated as 171488 Units/L. Amphotericin-B and nalidixic acid showed an E_max_ of 1.49 log_10_ CFU/mL and 2.54 log_10_ CFU/mL, respectively with EC_50_ as 16.67 mg/L and 90.97 mg/L, respectively. Trimethoprim and azilocillin failed to kill *M*. *tuberculosis* even with the highest tested concentration of 32 mg/L for both the drugs.

**Fig 2:**
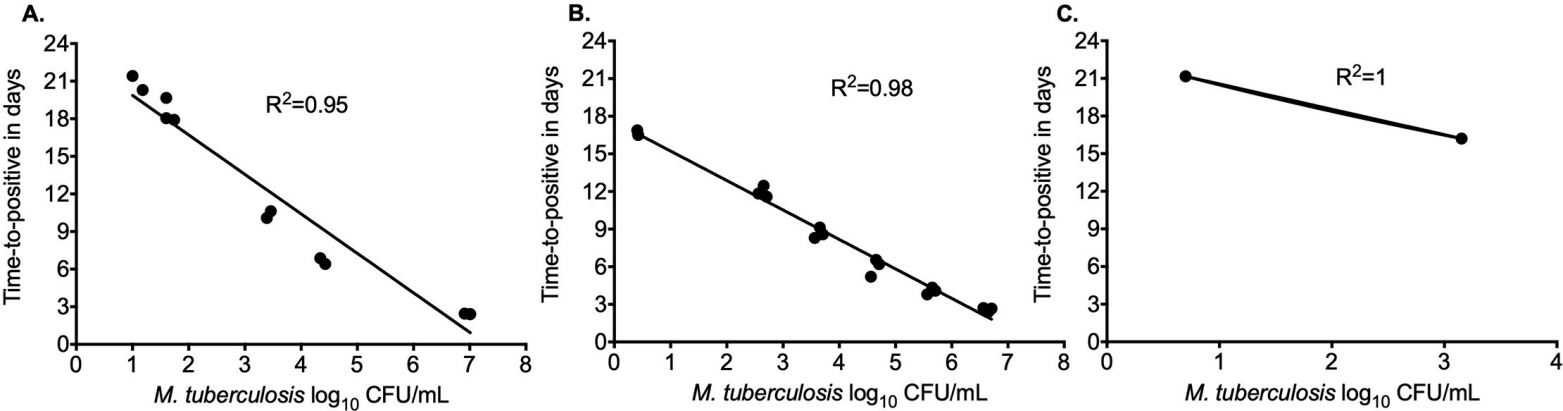
Correlation between the time-to-positive from MGIT and the CFU/mL. As shown by the R^2^, there was a good correlation between the *M*. *tuberculosis* bacterial burden recoded using the solid agar methods with the MGIT obtained time-to-positive for the **(A)** log phase growth, **(B)** semi-dormant bacilli, and **(C)** anaerobic cultures. However, the limit of detection was different between the metabolic populations.

Results of the experiments with three different *M*. *tuberculosis* metabolic populations to determine the correlation between the bacterial burden (CFU/mL) and the MGIT TTP are shown in **Fig 2A–2C**. The R^2^ for linear regression of log phase growth, semi-dormant bacilli and anaerobic population was 0.95, 0.98, and 1, respectively showing a good model fit with minimal bias. Next, we used the exponential growth model to calculate the doubling time of each metabolic population. It was calculated that in our experiments the doubling time for the log phase growth *M*. *tuberculosis* was 2.04 days, for the semi-dormant bacilli was 2.80 days, and was slowest for the anaerobic cultures as 6.37 days. The limits of detection by solid agar or TTP was 0.42 log_10_ CFU/mL.

## Discussion

Early detection and initiation of treatment is key to reducing the transmission of *M*. *tuberculosis*. Diagnosis is typically confirmed by detection of *M*. *tuberculosis* using microscopy in conjunction with the solid agar or liquid culture and or nucleic acid amplification.[12, 13] However, the culture confirmation could not be attained in a significant portion of patients (~ 20% to 30%) that are clinically diagnosed with tuberculosis.[14–16] Microbiologic growth is important to confirm the drug susceptibility testing to inform the clinicians on the drug selection to cater an effective treatment regimen. In the present study we determined the effect of the specimen processing method before inoculating the samples for isolation of the mycobacteria as well as effect of the antibiotic supplement and the growth characteristics of the different *M*. *tuberculosis* metabolic population that might affect the laboratory diagnosis.

First, we showed that NALC-NaOH method negatively affect the recovery of the *M*. *tuberculosis* in the samples. This is in agreement with previously published report where it averaged at 20% (range of 1.5% to 45%).[6] Due to this lower recovery rate, in recent years the focus has been shifted to use molecular diagnostic techniques. Second, we showed that the PANTA supplement used to prevent contamination with other bacteria and fungus in the MGIT liquid culture method, at the currently used antibiotic concentrations, does not negatively affect the mycobacteria recovery. Third, we showed that while polymyxin-B, trimethoprim, azilocillin do not have killing efficacy against *M*. *tuberculosis*, amphotericin-B and nalidixic acid were able to kill the *M*. *tuberculosis*. However, the concentrations needed to show the clinical efficacy of amphotericin-B and nalidixic acid are too high and will fall in the range of causing adverse effect. Fourth, we showed that the doubling time of *M*. *tuberculosis* in log phase or as semi-dormant bacilli was not significantly different from each other for our laboratory strains, however, the anerobic culture showed the slow doubling time and there was a 4 days lag before the anaerobic culture started showing growth units in the MGIT system. This means longer than routinely used 42 days time-in-protocol may be required in order to capture the slowest growing *M*. *tuberculosis* metabolic population in clinical samples.

Our study has limitations. We used only one concentration combination of NALC-NaOH in our decontamination protocol and the anaerobic culture condition could be considered lost their metabolic state as soon as the *M*. *tuberculosis* is collected for processing either for decontamination or inoculation into solid and liquid media for bacterial burden estimation. New methods need to be developed where the bacterial growth can be measured while maintaining the anaerobic state of *M*. *tuberculosis*.

In summary, samples processing method affect the recovery and in turn laboratory diagnosis of *M*. *tuberculosis*, PANTA at current antibiotic combination does not adversely affect *M*. *tuberculosis* growth, polymyxin-B and nalidixic acid have anti-tuberculosis efficacy at high concentrations, and the doubling time of different metabolic population should be considered when deciding the time-in-protocol for the MGIT system.
